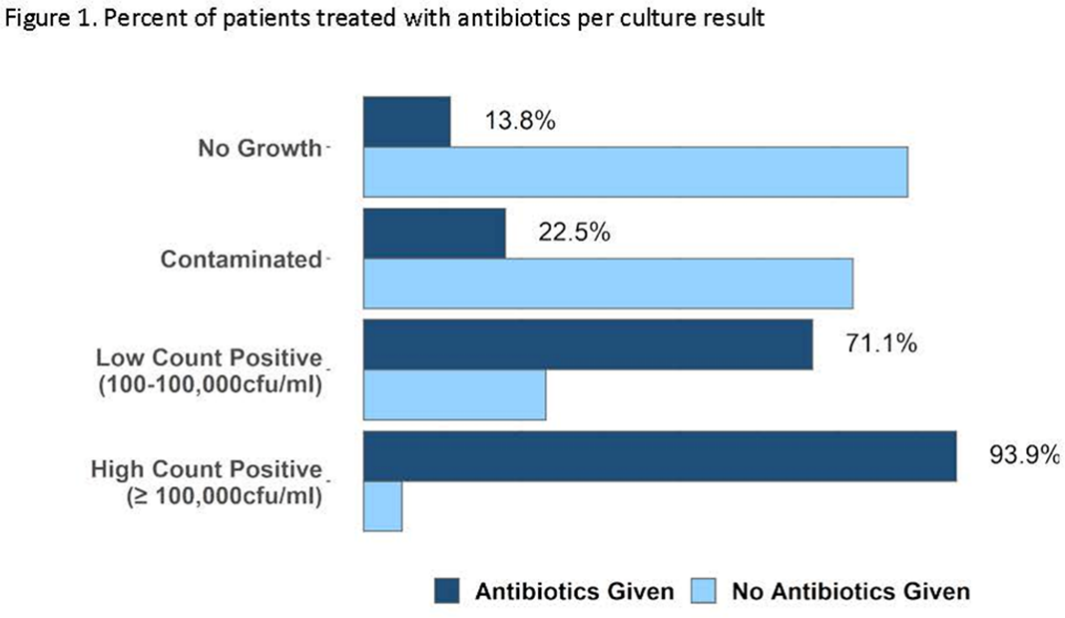# Optimizing Urine Collection Represents an Important Stewardship Opportunity in Primary Care

**DOI:** 10.1017/ash.2021.26

**Published:** 2021-07-29

**Authors:** Larissa Grigoryan, Jennifer Matas, Michael Hansen, Samuel Willis, Lisa Danek, Anna Katta, Kenneth Muldrew, Mohammad Zare, Forrest Hudson, Robert Atmar, Andrew Chou, Barbara Trautner

## Abstract

**Background:** Urine cultures are the most common microbiological tests in the outpatient setting and heavily influence treatment of suspected urinary tract infections (UTIs). Antibiotics for UTI are usually prescribed on an empiric basis in primary care before the urine culture results are available. However, culture results may be needed to confirm a UTI diagnosis and to verify that the correct antibiotic was prescribed. Although urine cultures are considered as the gold standard for diagnosis of UTI, cultures can easily become contaminated during collection. We determined the prevalence, predictors, and antibiotic use associated with contaminated urine cultures in 2 adult safety net primary care clinics. **Methods:** We conducted a retrospective chart review of visits with provider-suspected UTI in which a urine culture was ordered (November 2018–March 2020). Patient demographics, culture results, and prescription orders were captured for each visit. Culture results were defined as no culture growth, contaminated (ie, mixed flora, non-uropathogens, or ≥3 bacteria isolated on culture), low-count positive (growth between 100 and 100,000 CFU/mL), and high-count positive (*>*100,000 CFU/mL). A multivariable multinomial logistic regression model was used to identify factors associated with contaminated culture results. **Results:** There were 1,265 visits with urine cultures: 264 (20.9%) had no growth, 694 (54.9%) were contaminated, 159 (12.6%) were low counts, and 148 (11.7%) were high counts. Encounter-level factors are presented in Table 1. Female gender (adjusted odds ratio [aOR], 15.8; 95% confidence interval [CI], 10.21–23.46; *P* < .001), pregnancy (aOR, 13.98; 95% CI, 7.93–4.67; *P* < .001), and obesity (aOR, 1.9; 95% CI 1.31–2.77; *P* < .001) were independently associated with contaminated cultures. Of 264 patients whose urine cultures showed no growth, 36 (14%) were prescribed an antibiotic. Of 694 patients with contaminated cultures, 153 (22%) were prescribed an antibiotic (Figure 1). **Conclusions:** More than half of urine cultures were contaminated, and 1 in 5 patients were treated with antibiotics. Reduction of contamination should improve patient care by providing a more accurate record of the organism in the urine (if any) and its susceptibilities, which are relevant to managing future episodes of UTI in that patient. Optimizing urine collection represents a diagnostic stewardship opportunity in primary care.

**Funding:** This study was supported by the National Institute of Allergy and Infectious Diseases of the National Institutes of Health (grant no. UM1AI104681). The content is solely the responsibility of the authors and does not necessarily represent the official views of the National Institutes of Health.

**Disclosures:** None

**Table 1.**
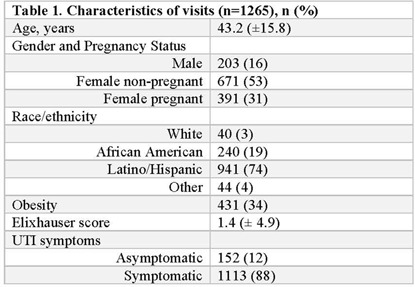

**Figure 1.**